# Human interferon-ω: an underappreciated type I interferon

**DOI:** 10.3389/fimmu.2026.1891351

**Published:** 2026-07-23

**Authors:** Ruyue Chen

**Affiliations:** Department of Nephrology and Immunology, Children’s Hospital of Soochow University, Suzhou, China

**Keywords:** anti-IFN-I autoantibodies, COVID-19, IFN-ω, inborn errors of immunity, type I interferons

## Abstract

Interferon-ω (IFN-ω) is a member of the human type I interferon family that has historically been overshadowed by IFN-α and IFN-β. Recent human “natural perturbations”, most notably selective neutralization of IFN-ω by autoantibodies in life-threatening viral infections, have renewed interest in this comparatively understudied cytokine and indicate that its antiviral activity may not always be fully compensated in defined clinical settings. This renewed focus has prompted reassessment of its single-gene organization, distinct antigenicity, intermediate receptor-binding kinetics, cellular sources, and regulatory mechanisms. In parallel, thymus-centered tolerance disorders, including autoimmune regulator (*AIRE*) deficiency, additional inborn errors of immune tolerance, and acquired “sick thymus” states, establish anti-IFN-ω autoantibodies as a stable, high-penetrance immunophenotype linking tolerance failure to durable cytokine-directed autoimmunity. Beyond antiviral defense, multi-omic studies in lupus-spectrum disease suggest tissue- and compartment-specific IFN-ω signals within broader type I interferon programs, yet endogenous IFN-ω remains rarely quantified with ligand-level resolution. This Review integrates current knowledge of IFN-ω from molecular regulation to human disease. Further progress will require subtype-resolved measurement, direct comparison with other type I interferons under matched conditions, and human genetic studies testing whether rare *IFNW1* variants contribute to severe viral disease.

## 1 Introduction

Interferons (IFNs) were first described in 1957, when Isaacs and Lindenmann identified a soluble factor induced by heat-inactivated influenza virus that conferred resistance to subsequent viral infection (1). Molecular cloning in the early 1980s established the classification of IFNs into type I and type II families, followed by the identification of type III interferons (IFN-λ) in the early 2000s (2–6). In humans, type I interferons (IFN-I) comprise 13 IFN-α subtypes together with IFN-β, IFN-ω, and the more tissue-restricted IFN-ϵ and IFN-κ, which are expressed predominantly in reproductive tract epithelium and skin keratinocytes, respectively (7–9). Despite their sequence diversity, all IFN-I signal through the shared IFNAR1/IFNAR2 receptor complex, activating Janus kinase 1 (JAK1) and tyrosine kinase 2 (TYK2), followed by phosphorylation of signal transducer and activator of transcription (STAT) proteins and induction of interferon-stimulated genes (ISGs) (10). Canonically, STAT1, STAT2, and interferon-regulatory factor 9 (IRF9) form the interferon-stimulated gene factor 3 (ISGF3) complex, which binds IFN-stimulated response elements (ISREs) to induce classical antiviral genes such as *OAS* and *MX1*, whereas STAT1 homodimers engage gamma-activated sequences (GAS) to activate a partially overlapping transcriptional program enriched in immune regulatory and pro-inflammatory genes, including *IRF1* and *CXCL9* (10). Insufficient IFN-I activity predisposes humans to life-threatening viral infections, particularly involving the respiratory tract and central nervous system (CNS), whereas excessive or sustained IFN-I signaling underlies a broad spectrum of autoinflammatory and autoimmune disorders known as type I interferonopathies (11, 12). These observations indicate that human IFN-I activity must be maintained within a narrow functional range.

**Figure 1:**
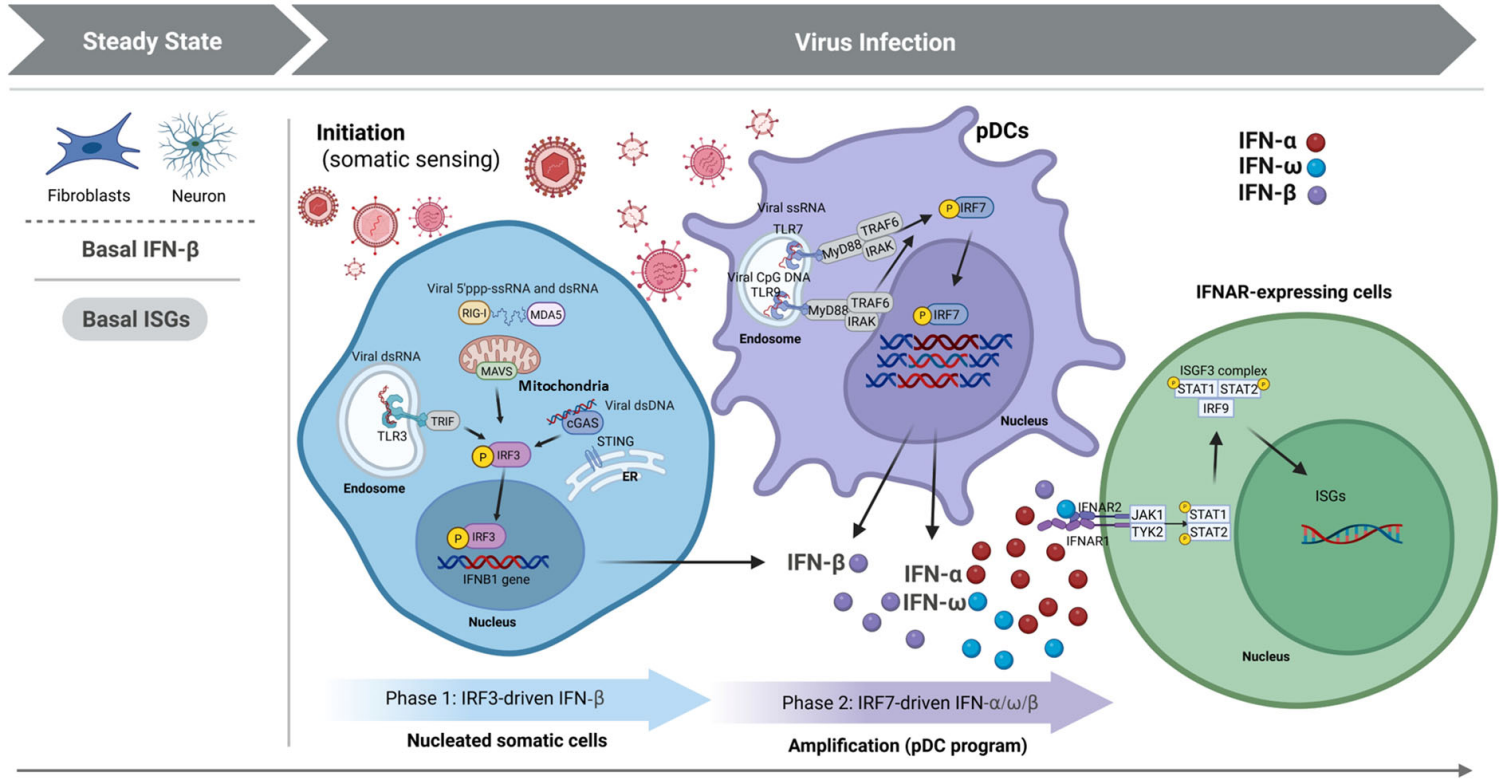
Cellular regulation and signaling of human type I interferons. At steady state, low basal IFN-β expression contributes to constitutive ISG activity in selected somatic cells. Following viral infection, cytosolic and endosomal nucleic-acid sensors activate IRF3-dependent IFN-β production in nucleated somatic cells, whereas TLR7/9-MyD88-IRF7 signaling supports rapid production of IFN-α, IFN-ω, and IFN-β by plasmacytoid dendritic cells. These ligands signal through IFNAR1/IFNAR2-associated TYK2 and JAK1 to activate the STAT1-STAT2-IRF9 complex and induce ISGs. The phases schematically represent response initiation and amplification.

Research on human IFN-I has historically focused predominantly on IFN-α and IFN-β. At steady state, IFN-β is expressed at low basal levels in some somatic cells such as fibroblasts and cortical neurons (13). Upon viral infection, nearly all nucleated cells rapidly induce IFN-β through innate sensing of pathogen-associated molecular patterns, thereby establishing an immediate antiviral state. This early IFN-β response sustains IFNAR signaling and induces IRF7, which in turn drives secondary amplification of IFN-α expression. As infection progresses, cooperative activation by IRF3 and IRF7 reinforces a positive-feedback loop that further amplifies IFN-α/β production, promoting viral clearance while also increasing pro-inflammatory output (14). (**Figure 1**) IRF3 is constitutively expressed in most cells, enabling widespread IFN-β production upon infection, whereas IRF7 is itself IFN-inducible and required for expression of most IFN-α subtypes, resulting in relatively limited IFN-α production in many cell types (15). However, plasmacytoid dendritic cells (pDCs) constitutively express high levels of IRF7 with heightened protein stability, enabling robust production of the full spectrum of IFN-α subtypes (15). Human IFN-ω remains considerably less well characterized, with much of the earlier evidence derived from animal studies or from experimental systems that did not examine IFN-ω as an individual ligand (16, 17). The identification of neutralizing anti-IFN-ω autoantibodies in patients with life-threatening viral infections, including severe COVID-19 pneumonia, has renewed interest in its human biology (18–23). These natural perturbations suggest that IFN-ω activity may not always be fully compensated by other IFN-I ligands in defined clinical settings, but do not establish a universally unique signaling or functional program. By integrating these human observations with molecular, cellular, and signaling evidence, this Review reassesses IFN-ω biology and the disease contexts in which its activity may become clinically consequential.

## 2 Decoding IFN-ω mechanisms

### 2.1 Human specificity and evolutionary conservation

Human genetics and serology provide direct *in vivo* evidence for IFN-ω biology, complementing decades of subtype-centered IFN-I research. The “human specificity” of IFN-ω is twofold, reflecting both its genomic organization within the human IFN-I locus and the marked divergence of IFN-ω repertoires across species. In humans, IFN-I genes are clustered on chromosome 9p21.3 and comprise 17 functional genes together with multiple pseudogenes, collectively encoding 16 distinct proteins: IFN-β, IFN-ω, IFN-ϵ, IFN-κ, and 12 IFN-α subtypes (*IFNA1* and *IFNA13* encode identical mature proteins and are therefore typically counted as a single IFN-α1) (24). (**Table 1**) Within this locus, *IFNW1* is retained as a single functional gene (with multiple pseudogenes), in sharp contrast to the expanded *IFNA* cluster. Human IFN-ω was initially considered part of an IFN-α subfamily and termed IFN-α subclass II (IFN-αII), sharing ~60% sequence identity with IFN-αs (versus ~31-38% between IFN-β and IFN-α, and ~76-96% among IFN-α subtypes) (24, 25). However, the absence of antigenic cross-reactivity between IFN-α and IFN-ω provided early support for classifying IFN-ω as a distinct IFN-I member (25). Structurally, mature IFN-ω is longer (174 aa) than IFN-β and most IFN-α proteins (~166 aa), consistent with a C-terminal extension (24). IFN-ω is also among the IFN-I that can be N-glycosylated (at N78, analogous to IFN-β N80), providing a structural layer that may influence stability, receptor engagement, or pharmacologic behavior (24).

**Table 1:** Human type I interferons: receptor binding and genetic constraint.

Protein	Principal cellular sources	IFNAR1 KD (μM)	IFNAR2 KD (nM)	Gene	Human genetic constraint (CoNeS)	Homozygous pLOF variants (gnomAD v4.1.1)
IFN-α1	pDC-dominant leukocytes	0.76	220	IFNA1/IFNA13	-0.15/-0.02	
IFN-α2 (α2a/α2b)	pDC-dominant leukocytes	3.5/3.8	1.7/1.3	IFNA2	1.19	
IFN-α4	pDC-dominant leukocytes	3	2.1	IFNA4	1.56	
IFN-α5	pDC-dominant leukocytes	0.94	3.8	IFNA5	1.14	p.Gln115Ter (n=40)
IFN-α6	pDC-dominant leukocytes	0.83	0.6	IFNA6	0.69	p.Gln85Ter (n=1)
IFN-α7	pDC-dominant leukocytes	1.3	0.7	IFNA7	1.72	p.Arg56Ter (n=2)
						p.Asn34Ter (n=1)
IFN-α8	pDC-dominant leukocytes	0.53	1.6	IFNA8	0.38	
IFN-α10	pDC-dominant leukocytes	4.3	0.4	IFNA10	2.97	p.Cys20Ter (n=47,160)
						p.Gln125Ter (n=38)
						p.Gln85Ter (n=14)
IFN-α14	pDC-dominant leukocytes	0.68	0.7	IFNA14	1.34	
IFN-α16	pDC-dominant leukocytes	1.1	1.1	IFNA16	1.75	
IFN-α17	pDC-dominant leukocytes	5	0.4	IFNA17	1.04	p.His57GlnfsTer2 (n=1,149)
IFN-α21	pDC-dominant leukocytes	1.25	5	IFNA21	2.21	p.Glu126Ter (n=2)
IFN-β	broad somatic cells	0.1	0.1	IFNB1	1.28	p.Arg186Ter (n=4)
IFN-ω	pDCs; monocytes	1	2	IFNW1	0.72	
IFN-ϵ	reproductive tract epithelium	2.2	70	IFNE	0.43	p.Gln71Ter (n=2,126)
						p.Met84IlefsTer53 (n=1)
IFN-κ	keratinocytes	0.34	21	IFNK	1.32	p.Trp13PhefsTer4 (n=912)
						p.Gln70Ter (n=3)
						p.Ala86ProfsTer31 (n=1)

Summary of human type I interferon proteins, their principal cellular sources, receptor-binding affinities, corresponding genes, consensus negative selection scores (CoNeS), and homozygous predicted loss-of-function (pLOF) variants reported in gnomAD v4.1.1. KD denotes the equilibrium dissociation constant; lower values indicate higher receptor-binding affinity. Receptor-binding values were compiled from published structural and biochemical studies (24), and CoNeS values from gene-level negative-selection analyses (31, 32). *IFNA1* and *IFNA13* encode the same mature IFN-α1 protein. Values shown for IFN-α2 correspond to IFN-α2a and IFN-α2b, respectively. pLOF entries show the protein consequence followed by the number of homozygotes in parentheses; blank cells indicate that no homozygous pLOF variant was included in the current summary.

Crucially, “IFN-ω” is not equivalent across species. Much of the earlier IFN-ω literature derives from non-primate mammals (notably livestock and companion animals), in which comparative genomics has revealed marked variation, including multigene IFN-ω families in several lineages, an architecture fundamentally different from the single-gene human *IFNW1* context (26, 27). Genome-wide surveys suggest that reptiles and birds typically encode a single IFN-ω-like gene, whereas numerous ungulates and bat species carry expanded IFN-ω repertoires, consistent with lineage-specific duplication (28). Phylogenetic analyses further indicate independent IFN-ω expansions across ungulate species, often forming one or two major species-restricted clusters; cattle and pigs exemplify this pattern, with 11–25 IFN-ω coding genes plus additional pseudogenes, representing several-fold more coding genes than in humans (28). Consistent with such divergence, IFN-ω can show partial cross-species antiviral activity, but responsiveness is often reduced across more distantly related species, arguing against universal interchangeability across mammals (26). In this sense, IFN-ω captures both species-specific diversification and partial cross-species conservation: IFN-ω repertoires diverge markedly across lineages, yet antiviral activity can remain measurable across multiple mammalian hosts (26, 28). Notably, IFN-ω is unevenly distributed across mammals and has not been identified in mice or canines in commonly used genome annotations, limiting the direct utility of classic murine models for human IFN-ω biology (26, 29). These cross-species discontinuities motivate examining whether *IFNW1* is genetically constrained in human populations.

Evolutionary genetic analyses place *IFNW1* within a relatively constrained subset of human IFN-I genes, alongside *IFNB1*, *IFNK*, and selected *IFNA* loci (e.g., *IFNA2*, *IFNA5*, *IFNA21*), based on the low frequency of amino-acid-altering variants and evidence of purifying selection on its coding sequence (30). By contrast, selective constraint across the *IFNA* family is heterogeneous: several *IFNA* genes (e.g., *IFNA6*, *IFNA8*, *IFNA13* and *IFNA14*) appear strongly constrained, whereas others tolerate common nonsynonymous variants (e.g., *IFNA1*, *IFNA4*, *IFNA7*, *IFNA10*, *IFNA16* and *IFNA17*), and homozygous nonsense alleles have been observed in selected interferon genes, including *IFNA10* and *IFNE* (30). Subsequent gene-level negative-selection analyses, including the consensus negative selection score (CoNeS), place *IFNW1* in an intermediate position within the broader interferon constraint spectrum rather than among the most absolutely constrained human genes (31, 32). (**Table 1**) Thus, genetic constraint supports the biological relevance of IFNW1 but cannot, by itself, establish the pathogenicity of an individual rare variant. Population-genetic analyses have also identified deviations in the *IFNW1* allele-frequency spectrum in Europeans (30). However, these neutrality-test signals neither demonstrate that clinically relevant IFNW1 variants are geographically restricted nor establish that rare variants are uniformly distributed across global populations. Under stringent criteria, strong geographically restricted recent positive selection was not a dominant feature of *IFNW1* or of the IFN-I family more broadly; the clearest population-restricted adaptive signals were instead observed among type III interferons (IFN-λ) (30). The ancestry-specific distribution and clinical penetrance of rare *IFNW1* variants therefore remain insufficiently defined. These uncertainties motivate ancestry-diverse sequencing of patients with otherwise unexplained severe viral infections, coupled with functional testing of rare candidate *IFNW1* variants.

### 2.2 Cellular sources and regulatory inputs

As early as 1999, pDC precursors were identified as the principal type I interferon-producing cells (natural IFN-I producing cells) in human blood, generating ~200 to 1,000-fold more IFN-I than other circulating leukocytes upon microbial challenge, although these early studies largely measured IFN-α-dominant activity (33). Direct evidence linking IFN-ω to pDC biology emerged soon thereafter. In 2002, IFN-ω production was shown to be strongly pDC-dependent: following stimulation with TLR7 agonists or herpes simplex virus (HSV), IFN-ω was detectable in stimulated peripheral blood mononuclear cells (PBMCs) but absent in pDC-deficient PBMCs, and IFN-ω output was enriched ~3 to 8-fold in pDC-enriched cultures compared with bulk PBMCs (34). A similar bias was observed for IFN-α, as TLR7 agonist-treated pDC-enriched cultures produced ~2 to 20-fold more IFN-α than similarly treated PBMCs across the same dose range, consistent with a shared pDC-centered induction program (34). More recent *ex vivo* profiling in human PBMCs showed that CpG-A (TLR9-linked) and cGAMP (STING-linked) each elicit robust IFN-ω secretion at levels comparable to IFN-α2 and IFN-β; mechanistically, IFN-ω production mapped to distinct upstream sensors in different cell types: pDC depletion abolished the CpG-A-driven response, monocyte depletion abolished the cGAMP-driven response, and dual depletion abrogated IFN-ω induction by either stimulus (35). This division of labor mirrored the patterns observed for IFN-α2 and IFN-β outputs, identifying pDCs and monocytes as the dominant sources of IFN-ω in PBMCs and supporting IFN-ω production on par with its “sister” IFN-I ligands (35). By contrast, B cell depletion did not measurably alter IFN-I levels in this setting (35).

This framework is consistent with established principles of human IFN-I regulation. Compared with myeloid dendritic cells (mDCs), pDCs exhibit prominent endosomal expression of TLR7 and TLR9, which are specialized for sensing viral RNA- and DNA-derived nucleic acids (36). In pDCs, activation of TLR7 and TLR9 signals through the MyD88-IRF7 axis and supports rapid, high-level IFN-I production, whereas in most nucleated somatic cells early IFN-β induction depends more strongly on IRF3, with subsequent autocrine IFNAR signaling required to induce IRF7 and amplify IFN-α/β production (36). Because pDCs constitutively express high levels of IRF7, they can mount rapid and robust IFN-α output with reduced dependence on prior IFN-β feedback (36). These properties established pDCs as the canonical platform for dissecting IFN-α biology, whereas the upstream logic and cellular sources governing IFN-ω induction remain less systematically defined. By comparison, monocytes can represent a dominant source of IFN-α downstream of cytosolic DNA sensing through the cGAS-STING pathway in whole PBMC systems (37). In this setting, cGAMP-induced IFN-α tended to be higher in older individuals and correlated with increased monocyte numbers with age, whereas no major sex bias was detected (37). This contrasts with TLR7/9-driven IFN-α responses, which show consistently stronger responses in females across ages (37). Although much of this regulatory architecture has been defined in IFN-α-focused studies, it likely provides an important framework for IFN-ω, suggesting that a similar division of labor between endosomal TLR7/9-driven pDC programs and cytosolic sensing in monocytes may help define when, where, and through which sensors IFN-ω is induced in humans. Recent work identified IFN-ω as one component of a sterol regulatory element-binding protein 1 (SREBP1)-dependent noncanonical IFN-I program induced by cholesterol restriction. In responsive human cell models, statin treatment increased *IFNW1* expression and IFN-ω protein production, SREBP1 directly activated the *IFNW1* promoter, and *IFNW1* knockout impaired subsequent RIG-I and MDA5 upregulation (38).

### 2.3 Signaling dynamics and ISG programs

Although IFN-ω engages the shared IFNAR1/2 receptor complex, subtype-specific differences in receptor-binding affinity, kinetics, and ternary-complex stability can quantitatively shape the magnitude and duration of JAK-STAT signaling, thereby influencing downstream ISG programs. IFN-I ligands typically bind IFNAR1 with micromolar affinity and IFNAR2 with nanomolar affinity, reflecting highly asymmetric receptor engagement (39). However, receptor-binding affinities vary substantially across subtypes. IFN-β is the highest-affinity ligand interacting with IFNAR1 at ~0.1 μM and IFNAR2 at ~0.1 nM, whereas IFN-ω displays intermediate affinities (~1 μM for IFNAR1 and ~2 nM for IFNAR2) that fall within the broader range observed across IFN-α subtypes (24, 39). (**Table 1**) Kinetic parameters provide additional resolution for these subtype-specific differences. IFN-I ligands generally associate rapidly with IFNAR2 (ka ~10^6^-10^7^/M/s), whereas association with IFNAR1 is comparatively slower (ka ~2×10^5^/M/s) and shows relatively limited variation across subtypes (40). For IFNAR2, IFN-β shows the fastest association rate (ka ~1×10^7^/M/s), whereas IFN-ω occupies an intermediate position (ka ~6×10^6^/M/s) and representative IFN-α subtypes such as IFN-α2 show somewhat lower rates (ka ~3×10^6^/M/s) (40). Dissociation kinetics follow a similar hierarchy: IFN-β dissociates most slowly from IFNAR2 (kd ~0.001/s), whereas IFN-ω shows an intermediate rate (kd ~0.008/s), and IFN-α2 dissociates more rapidly (kd ~0.015/s) (40). Accordingly, receptor-binding lifetimes also differ, with IFN-β remaining bound to IFNAR2 for ~1000s, whereas IFN-ω and IFN-α2 exhibit lifetimes on the order of ~100s, consistent with more sustained receptor engagement and prolonged downstream signaling by IFN-β (39). These quantitative differences occur within broadly conserved ternary-complex architectures across IFN-I ligands. Structural comparisons of IFN-ω- and IFN-α2-bound IFNAR1/2 complexes show highly similar docking modes and largely conserved receptor-contacting residues, supporting the view that subtype-specific functional differences reflect quantitative tuning of receptor engagement rather than fundamentally distinct three-dimensional binding modes (41).

Signal propagation downstream of IFN-ω proceeds through the canonical IFNAR-associated kinase module. The intracellular domain of IFNAR1 associates with TYK2, whereas that of IFNAR2 associates with JAK1; upon ligand-induced assembly of the extracellular signaling complex, these kinases initiate phosphorylation cascades dominated by STAT activation (41). In reporter-based assays, IFN-ω, like IFN-α and IFN-β, activates STAT/IRF-linked readouts (e.g., SEAP reporters reflecting STAT1-STAT2-IRF9/ISGF3 activity), whereas NF-κB-linked reporter activity remains low under the same conditions, indicating predominant engagement of the classic IFN-JAK-STAT axis (35). At the transcriptional level, IFN-ω elicits robust antiviral ISG programs in epithelial cells. In Calu-3 cells, IFN-ω induces a broad antiviral gene module that closely overlaps with IFN-α2, including *MX1*, *ISG15*, *IFI27*, and *MX2*, whereas IFN-ϵ fails to generate a comparably strong antiviral signature (35). In the same work, sustained high-dose IFN-ω exposure before and after infection fully suppressed severe acute respiratory syndrome coronavirus 2 (SARS-CoV-2) replication, linking durable ISG induction to a functionally effective antiviral state when IFN-ω is present at sufficient concentration and duration (35). Beyond epithelial antiviral programs, IFN-ω can also modulate cytotoxic lymphocyte circuitry. In human CD8+ T cell subsets, IFN-ω triggers rapid STAT1 and STAT4 phosphorylation with kinetics similar to IFN-α2 and IFN-β, increases expression of transcription factor T-bet, and enhances cytolytic effector functions; its effect on naïve CD8+ T cell proliferation is broadly comparable to those of IFN-α2/β (35). Taken together, current evidence does not support a distinct receptor or an entirely separate signaling pathway for IFN-ω. Any apparent incomplete compensation is likely to be context-dependent, reflecting differences in cellular source, tissue distribution, timing and duration of production, receptor-binding kinetics, and selective neutralization among IFN-I ligands *in vivo*. Whether IFN-ω exerts genuinely ligand-selective effects beyond quantitative differences in shared IFNAR signaling remains unresolved and requires direct comparisons under matched experimental conditions.

## 3 Revealing IFN-ω in antiviral defense

### 3.1 COVID-19 defines the clinical relevance of IFN-ω

**Figure 2:**
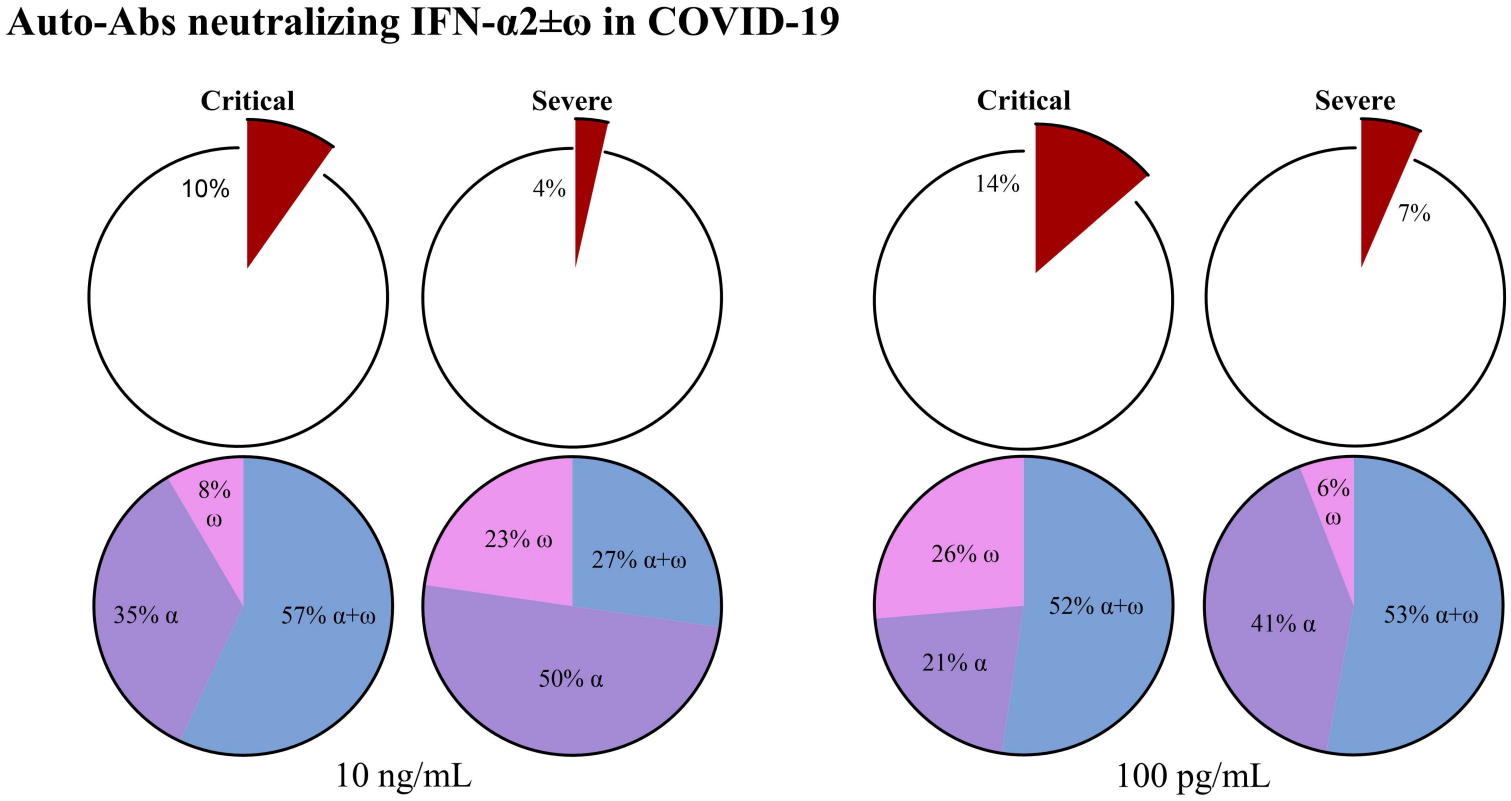
Neutralizing autoantibodies against IFN-α2 ± IFN-ω in COVID-19. The upper panels show the prevalence of neutralizing autoantibodies in patients with critical or severe COVID-19 at 10 ng/mL and 100 pg/mL, and the lower panels show their specificity distribution among neutralization-positive patients (18).

The SARS-CoV-2 pandemic provided the first population-level evidence that pre-existing neutralizing autoantibodies against IFN-I (predominantly IFN-α and IFN-ω, and less frequently IFN-β) can predispose to life-threatening viral disease (18, 42). At the stringent 10 ng/mL neutralization threshold, these autoantibodies were detected in ~10% of patients with critical COVID-19 and 4% of those with severe disease, compared with 0.37% of controls (18). Among seropositive patients, combined IFN-α2/ω neutralization predominated in critical disease, whereas IFN-ω-only neutralization represented a larger proportion of severe than critical cases (18). Lowering the assay concentration to 100 pg/mL increased the proportion of patients identified and altered the apparent specificity distribution, including a larger IFN-ω-only fraction in critical disease (18). Across thresholds, ~15% of anti-IFN-α2/ω-positive patients neutralized IFN-ω without detectable IFN-α2 neutralization (18). In the overall cohort of patients with critical COVID-19, IFN-ω neutralization (alone or combined) was detected in 6.4% and 10.7% at 10 ng/mL and 100 pg/mL (OR = 13.0 [95% CI, 4-38] and 13.0 [95% CI, 7-23], versus asymptomatic/mild infection), whereas IFN-ω-only neutralization accounted for 0.8% and 3.6% of critical cases at these thresholds (OR = 3.0 [95% CI, 0.9-10] and 6.0 [95% CI, 3-12]) (18). (**Figure 2**) Notably, sera neutralizing IFN-ω did not cross-neutralize the other 11 IFN-α subtypes, whereas anti-IFN-α2-positive sera frequently neutralized multiple IFN-α subtypes, indicating a distinct antigenic profile for IFN-ω (18). Anti-IFN-β autoantibodies were comparatively rare (1.3% in critical cases and absent in severe disease), and 78.3% of anti-IFN-β-positive individuals lacked reactivity against IFN-α2/ω (18).

**Figure 3:**
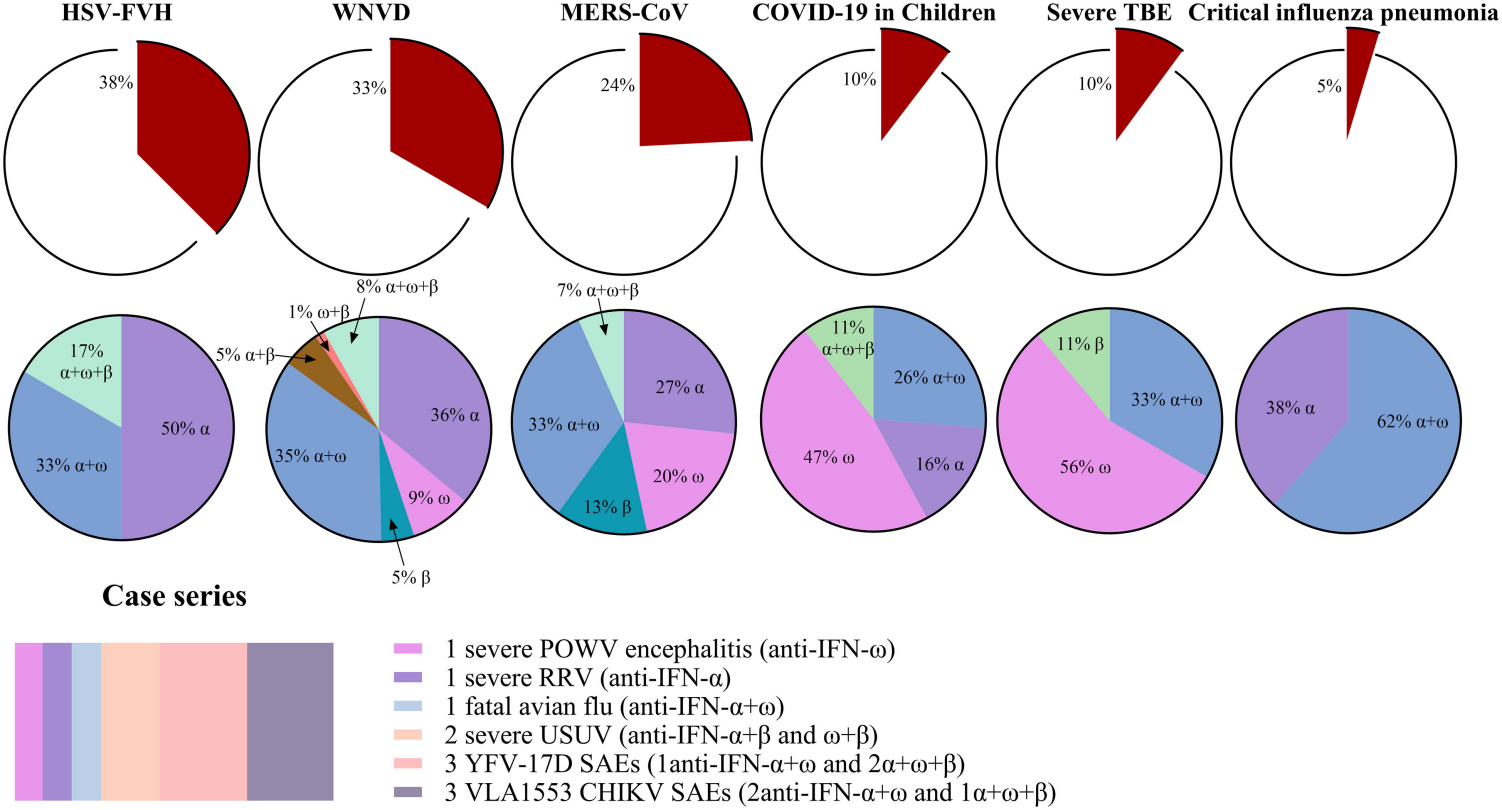
Neutralizing anti-IFN-I autoantibodies across severe viral diseases. The upper panels show the prevalence of neutralizing anti-IFN-I autoantibodies in each disease setting, and the lower pie charts show their specificity distribution among neutralization-positive patients (19–23, 47, 48, 50–52). The lower panel summarizes selected case reports and small series. Neutralization thresholds varied across studies; the data are therefore presented descriptively as reported in the original publications. HSV-FVH, herpes simplex virus-associated fulminant viral hepatitis; WNVD, West Nile virus disease; MERS-CoV, Middle East respiratory syndrome coronavirus; TBE, tick-borne encephalitis; POWV, Powassan virus; RRV, Ross River virus; USUV, Usutu virus; YFV-17D, yellow fever vaccine 17D; CHIKV, chikungunya virus; SAE, severe adverse event.

The pediatric cohort showed a different autoantibody-specificity profile from that observed in adult COVID-19 cohorts. Neutralizing anti-IFN-I autoantibodies were detected in ~10% of unvaccinated children hospitalized with COVID-19 pneumonia. Among autoantibody-positive children, 74% had critical, 21% severe, and 5% moderate disease (22). IFN-ω-only neutralization was the most frequent specificity (~47%), compared with 26% dual IFN-α2/ω, 16% IFN-α2-only, and 11% triple IFN-α2/β/ω neutralization (22). (**Figure 3**) Neutralization at high concentrations of IFN-α2/ω was associated with higher odds of life-threatening pneumonia [OR 12.9 (95% CI, 4.6–35.9)] than neutralization at low concentrations [OR 5.5 (95% CI, 3.1–9.6)] (22). However, breadth and specificity were not equivalent: IFN-ω-only neutralization showed comparatively modest risk [OR 2.6 (95% CI, 1.2-5.3)], whereas IFN-α2-only neutralization was associated with much higher odds (OR 67.6 [95% CI, 5.7-9,196.6]) (22). Population data further showed that neutralizing autoantibodies to IFN-α2 or IFN-β are exceedingly rare in children from the general population (0.17% and 0.04%, respectively), whereas anti-IFN-ω autoantibodies are more frequent (~0.35% at 10 ng/mL, rising to ~2% at 100 pg/mL) (22). The prevalence of neutralizing anti-IFN-ω autoantibodies declines slightly through middle age and rises after 65 years (~2.5-fold), a smaller age effect than observed for anti-IFN-α2 autoantibodies (≈8-fold overall, reaching ~10-fold in men) (22). Accordingly, neutralization of IFN-ω at 100 pg/mL is more common in children (~2%) than in adults (~0.9%) and remains ~2% in the elderly (22).

Extending beyond blood, neutralizing anti-IFN-I autoantibodies were detected in bronchoalveolar lavage fluid from 13% of patients with life-threatening COVID-19 pneumonia, with autoantibody-positive patients showing IFN-ω-only (17%), IFN-α-only (28%), dual IFN-α/ω (46%), and triple IFN-α/β/ω (6%) reactivity (43). These human “natural perturbations” are complemented by mucosal and *ex vivo* data further supporting IFN-ω as an active component of antiviral defense. Patients without autoantibodies generally mounted strong nasal IFN-I/ISG responses at high viral loads, whereas those with anti-IFN-I autoantibodies showed blunted responses irrespective of viral load, except when neutralization was restricted to IFN-α2, consistent with possible compensation by IFN-ω (44). Age further shaped this landscape. At baseline, nasopharyngeal IFN-α, IFN-ω, and IFN-ϵ transcripts in young adults were intermediate between pediatric and older adults, and declined with age, whereas IFN-β resembled older adults (45). Upon SARS-CoV-2 infection, IFN-I/ISG programs were induced across age groups, but IFN-α and IFN-β varied little with age, while IFN-ω and IFN-ϵ remained inversely correlated with age (45). Across cohorts, SARS-CoV-2 positivity was associated with higher IFN-α, IFN-ω and ISG15 expression, and IFN-α/ω transcript levels were modestly higher in symptomatic than asymptomatic children and in hospitalized than non-hospitalized adults (45). Mechanistically, IFN-ω drove an IFN-α2-like antiviral transcriptional program, and under sustained high-dose exposure before and after infection, fully suppressed SARS-CoV-2 replication with strong induction of *MX1* and *OAS2* (35). IFN-ω also showed IFN-α2-like STAT1/STAT4 phosphorylation and promoted T-bet expression and CD8^+^ T cell effector functions, with effects broadly comparable to IFN-α2/β (35). Exocyst complex component 2 (EXOC2), a core exocyst subunit involved in vesicle tethering, was recently proposed as a host factor for SARS-CoV-2 infection, and *EXOC2* knockdown reduced infection efficiency in human cellular models by regulating *IFNW1* expression, supporting a mechanistic link between exocyst-dependent trafficking and IFN-ω-related antiviral responses (46). (**Table 2**).

**Table 2:** Clinical settings linking IFN-ω to human disease.

Clinical setting	IFN-ω-related finding	Specimen	Main readouts	References
Pneumotropic viral infections				
Life-threatening COVID-19 pneumonia (SARS-CoV-2)	Anti-IFN-ω±α/β autoAbs	Blood; airway (BAL/nasal mucosa)	Neutralization; mucosal ISGs	(18, 22, 43–45)
Severe MERS pneumonia (MERS-CoV)	Anti-IFN-ω±α/β autoAbs	Blood	Neutralization	(23)
Critical influenza pneumonia (Seasonal influenza)	Anti-IFN-ω+α autoAbs	Blood	Neutralization	(47)
Fatal avian influenza pneumonia (H5N1)	Anti-IFN-ω+α autoAbs	Blood	Neutralization	(48)
Neurotropic viral infections				
Severe tick-borne encephalitis (TBE)	Anti-IFN-ω±α autoAbs	Blood	Neutralization	(21)
Neuroinvasive West Nile virus disease (WNVD)	Anti-IFN-ω±α/β autoAbs	Blood; CSF	Neutralization	(19)
Powassan virus encephalitis (POWV)	Anti-IFN-ω autoAbs	Blood	Neutralization	(20)
Severe Usutu virus infection (USUV)	Anti-IFN-ω+β autoAbs	Blood	Neutralization	(20)
Attenuated vaccine-SAEs				
Yellow fever vaccine-SAEs (YFV-17D)	Anti-IFN-ω+α/ω+α+β autoAbs	Blood	Neutralization	(51)
Chikungunya vaccine-SAEs (VLA1553 CHIKV)	Anti-IFN-ω+α/ω+α+β autoAbs	Blood; CSF	Neutralization	(52)
Other viral infections				
HSV-associated fulminant viral hepatitis (HSV-FVH)	Anti-IFN-ω+α/ω+α+β autoAbs	Blood	Neutralization	(50)
Human immunodeficiency virus (HIV)	Anti-IFN-ω autoAbs	Blood	Neutralization	(53)
Immune dysregulation syndromes related to anti-IFN-ω autoAbs				
APS-1 (*AIRE* deficiency)	Near-universal anti-IFN-ω autoAbs (often with anti-IFN-α)	Blood	Serology; neutralization	(56, 57)
Alternative NF-κB pathway defects (*NFKB2/RELB/NIK*)	Anti-IFN-ω±α autoAbs	Blood	Serology ± neutralization	(62)
Hypomorphic *RAG1/2* CID	Anti-IFN-ω±α autoAbs	Blood	Serology ± neutralization	(63)
IPEX (FOXP3)	Anti-IFN-ω±α autoAbs	Blood	Serology ± neutralization	(67)
Thymoma/myasthenia gravis	Anti-IFN-ω±α autoAbs	Blood	Serology; neutralization	(72, 73)
Systemic autoimmunity associated with IFN-ω signals				
Systemic lupus erythematosus (SLE)	IFN-ω protein signal (association)	Blood	Proteomics; ISG score	(78)
Discoid lupus (skin)	*IFNW1*-response signature enriched	Skin	Transcriptomics	(80)
Lupus nephritis (class III/IV)	*IFNW1*-response signature enriched	Kidney (glomeruli, tubulointerstitium)	Transcriptomics	(80)
Dermatomyositis (skin)	*IFNW1* co-induction with IFN-α subtypes	Skin	Transcriptomics	(84)

Literature-based summary of clinical settings in which IFN-ω-related autoantibodies, protein signals, or transcriptional signatures have been reported. Viral-disease entries refer to functional neutralizing autoantibodies unless otherwise indicated. In immune dysregulation syndromes, serologic detection and/or functional neutralization are specified in the readout column. Transcriptomic entries indicate IFNW1-related or IFN-ω-associated signatures rather than direct measurement of endogenous IFN-ω protein. APS-1, autoimmune polyendocrine syndrome type 1; BAL, bronchoalveolar lavage; CID, combined immunodeficiency; CSF, cerebrospinal fluid; HSV-FVH, herpes simplex virus-associated fulminant viral hepatitis; IPEX, immune dysregulation, polyendocrinopathy, enteropathy, X-linked; ISG, interferon-stimulated gene; MERS-CoV, Middle East respiratory syndrome coronavirus; POWV, Powassan virus; SAE, severe adverse event; TBE, tick-borne encephalitis; USUV, Usutu virus; WNVD, West Nile virus disease; YFV-17D, yellow fever vaccine 17D; CHIKV, chikungunya virus.

### 3.2 Extension to other severe viral infections

COVID-19 revealed that pre-existing neutralizing autoantibodies against IFN-α/ω can function as a “natural knockout” of this arm of antiviral immunity, predisposing to life-threatening viral pneumonia. This framework extends to other severe viral infections, with pathogen- and tissue-specific patterns. In Middle East respiratory syndrome coronavirus (MERS-CoV) pneumonia, neutralizing anti-IFN-I autoantibodies were detected in 24% of patients, with a heterogeneous specificity distribution that included IFN-ω-only (20%), IFN-α-only (27%), IFN-β-only (13%), and broader co-neutralization patterns (33% IFN-α+ω and 7% IFN-α+ω+β) (23). The autoantibody profile differed from that observed in critical influenza pneumonia: IFN-ω-only neutralization was present in MERS-CoV pneumonia but absent in influenza, in which combined IFN-α/ω neutralization predominated (47). The absence of isolated IFN-ω neutralization and the predominance of combined IFN-α/ω blockade suggest that critical influenza is more commonly associated with broader disruption of the IFN-I response. This pattern is also consistent with a fatal case of H5N1 avian influenza pneumonia in which autoantibodies neutralized all 12 IFN-α subtypes at 1–10 ng/mL and IFN-ω at 100 pg/mL (48). (**Figure 3**) Functional data also support a role for IFN-ω in mucosal antiviral defense beyond coronaviruses. In guinea pigs, intranasal recombinant human IFN-ω rapidly induced Mx and OAS-1a, and inhibited 2009 pandemic A (H1N1) influenza to an extent comparable to IFN-α, with reduced lung viral titers from day 1 after exposure (49).

Among neurotropic flaviviruses, autoantibody profiles differed substantially by pathogen. IFN-ω-only neutralization predominated among seropositive patients with severe tick-borne encephalitis (TBE), whereas West Nile virus disease (WNVD) was characterized mainly by IFN-α-only and combined IFN-α/ω neutralization. In severe TBE, anti-IFN-I autoantibodies were detected in 10% of patients, and seropositive cases were dominated by IFN-ω-only neutralization (56%), with an additional 33% showing IFN-α+ω co-neutralization and 11% neutralizing IFN-β (21). Compared with the general population, anti-IFN-ω autoantibodies were associated with higher odds estimates than anti-IFN-α, with odds ratios of 12.3 (95% CI, 3.0-51.4) and 7.2 (95% CI, 2.1-24.4) at 10 ng/mL and 100 pg/mL, respectively, versus 8.1 (95% CI, 1.8-37.4) and 2.9 (95% CI, 0.6-13.5) for anti-IFN-α (21). This marked skew supports a clinically relevant contribution of IFN-ω in severe TBE. In WNVD, anti-IFN-I autoantibodies were even more prevalent, being detected in 33% of cases overall (36% in neuroinvasive disease and 23% in non-neurological disease), compared with 12% of outpatients with WNV fever and 3% of individuals with recent asymptomatic or mild infection (19). Among seropositive WNVD patients, neutralization patterns were dominated by IFN-α alone (36%) and IFN-α+ω co-neutralization (35%), whereas IFN-ω-only neutralization accounted for 9%; less frequent profiles included IFN-β alone (5%), IFN-α+β (5%), IFN-ω+β (1%), and triple IFN-α+ω+β neutralization (8%) (19). A similar distribution was observed across neuroinvasive subgroups (19). Rare case reports further broaden this spectrum. A patient with severe Powassan virus (POWV) encephalitis carried isolated neutralizing anti-IFN-ω autoantibodies (20). In addition, two patients with severe Usutu virus (USUV) infection harbored anti-IFN-I autoantibodies with distinct specificities and clinical presentations: one with meningitis neutralized IFN-α+β, whereas the other, presenting with myocarditis, systemic inflammatory response syndrome and acute renal failure, neutralized IFN-ω+β (20). (**Figure 3**).

Beyond pneumotropic and neurotropic syndromes, several additional settings further define the clinical boundaries of IFN-I dependence. In HSV-associated fulminant viral hepatitis, the phenotype is dominated by hepatic injury rather than classic neurotropism, and the absence of an IFN-ω-only pattern was consistent with an association with broader IFN-I disruption, with 33% of seropositive cases neutralizing IFN-α+ω and 17% neutralizing IFN-α+ω+β (50). Attenuated vaccine-associated severe adverse events (SAEs) similarly support breadth-dependent vulnerability. Among three yellow fever 17D vaccine SAEs, autoantibody specificities uniformly involved the IFN-α/ω axis: one case neutralized IFN-α+ω, and two neutralized IFN-α+ω+β (51). Clinically, these events were dominated by yellow fever vaccine-associated viscerotropic disease with hepatic involvement, and one case additionally developed meningoencephalitis (51). Similarly, among three VLA1553 chikungunya vaccine SAEs, two cases neutralized IFN-α+ω and one neutralized IFN-α+ω+β; notably, all developed encephalitic complications, consistent with an association between broad IFN-I neutralization and encephalitic complications following vaccination (52). (**Figure 3**) By contrast, anti-IFN-I autoantibodies were detected in only approximately 0.82% of people with HIV, a frequency comparable to that in the general population, and neutralizing activity was restricted to IFN-ω (53). Seropositive individuals nevertheless reported extensive histories of viral infections and virus-associated cancers, suggesting a potential link between anti-IFN-I autoantibodies and recurrent/persistent viral infections (53). Across diverse viral syndromes, functional anti-IFN-ω autoantibodies indicate that loss of IFN-ω activity can be clinically relevant in selected settings. Whether rare *IFNW1* variants similarly predispose to severe viral disease remains unknown and requires human genetic and functional investigation. (**Table 2**).

## 4 Expanding IFN-ω beyond antiviral function

### 4.1 Immune dysregulation syndromes related to anti-IFN-ω autoantibodies

Genetic defects in immune tolerance provide a prototypical setting in which anti-IFN-I autoantibodies arise as a stable, high-penetrance phenotype, exemplified by autoimmune polyendocrine syndrome type 1 (APS-1) caused by autoimmune regulator (*AIRE*) deficiency (54). In 2006, Meager et al. reported that APS-1 patients harbored high-titer neutralizing IgG against most IFN-α subtypes, with particularly strong reactivity to IFN-ω, whereas 23% also carried lower-titer anti-IFN-β antibodies (55). Subsequent studies confirmed near-universal neutralization of IFN-ω together with a high prevalence of anti-IFN-α2 autoantibodies, irrespective of clinical phenotype, disease duration, or *AIRE* genotype, establishing anti-IFN-I autoantibodies as a diagnostic hallmark of APS-1 (56). Anti-IFN-ω autoantibodies are often the most prevalent specificity in APS-1 cohorts (56–58), can be detected in the earliest available samples (55) and have been documented as early as 4 months of age in a presymptomatic infant (59). Mechanistically, AIRE is a central regulator of thymic immune tolerance, promoting ectopic expression of tissue-restricted antigens in medullary thymic epithelial cells (mTECs) for presentation to developing T cells (60, 61). Two non-mutually exclusive hypotheses have been proposed to explain the origin of anti-IFN-I autoantibodies (61). In a T cell-driven central tolerance model, thymic expression of IFNs normally contributes to deletion of IFN-reactive T cells, whereas defective thymic organogenesis or impaired AIRE expression permits their escape and subsequent autoimmunity. In a B cell-driven peripheral-break model, unusually high IFN levels in the thymus and/or peripheral tissues early in life, often in an infection-inflamed milieu, promote tissue damage and breach peripheral tolerance. Once established, these anti-IFN-I responses can remain durable throughout life (61).

Anti-IFN-I autoantibodies also occur in a broader spectrum of IEIs that converge on defective thymic tolerance through distinct but partially overlapping mechanisms. Particularly informative are defects affecting the alternative NF-κB pathway, including *NFKB2*, *RELB*, and *NIK*, which impair the development of AIRE-expressing mTECs and thereby compromise AIRE-dependent central tolerance (62). Complementing this stromal axis are disorders that perturb thymocyte development and selection. Biallelic hypomorphic *RAG1/2* variants associated with combined immunodeficiency have been reported in patients with anti-IFN-I autoantibodies, plausibly reflecting restricted TCR diversity and disrupted intrathymic T cell development that permits the escape of autoreactive clones (63–65). Additional perturbations of T cell signaling, including *JAK3*, *STAT1*, and *LCK* variants, further suggest that abnormalities in thymocyte-intrinsic signaling may impair intrathymic selection and contribute to tolerance failure (61, 66). Beyond defects in thymic epithelial or developmental compartments, a further layer of susceptibility arises from impaired immune regulation. Immunodysregulation polyendocrinopathy enteropathy X-linked (IPEX) syndrome, caused by deleterious hemizygous *FOXP3* variants, exemplifies how defective regulatory T cell differentiation can erode tolerance and permit anti-IFN-I autoreactivity (67, 68). Similarly, *CTLA4* haploinsufficiency and *IKZF2* deficiency further broaden this spectrum and have been linked with anti-IFN-α/ω autoantibodies in selected cohorts, highlighting the contribution of immune checkpoint pathways and regulatory T-cell programs to tolerance maintenance beyond classical thymic epithelial defects (66, 69, 70). At a more structural level, disorders associated with thymic structural abnormalities provide an additional perspective. X-linked dominant incontinentia pigmenti due to *NEMO* deficiency has been linked to anti-IFN-I autoantibodies in the context of thymic dysplasia, with neutralizing anti-IFN-α/ω detected in 36% of affected females, suggesting that disruption of thymic architecture itself can compromise the cellular niches required for effective tolerance induction (71). Despite their genetic heterogeneity, these IEIs converge on a common outcome: failure of thymus-centered tolerance programs, through either thymocyte-intrinsic or thymic stromal defects, that permits anti-IFN-I autoreactivity.

A parallel acquired counterpart is observed in selected adult-onset disorders with thymus-centered pathology. The prototypical acquired “sick thymus” setting comprises thymoma- and/or myasthenia gravis (MG)-related immune dysregulation, in which high-titer IgG autoantibodies that neutralize IFN-α2 and IFN-ω, frequently accompanied by anti-IL-12, are detectable in late-onset myasthenia gravis or thymoma alone and are most enriched when both conditions coexist (72). Consistently, one cohort showed that autoantibodies against IFN-ω, IFN-α2, and IFN-α8 occurred more often in APS-1 (100%) and MG with thymoma (73%) than in late-onset MG (39%) or early-onset MG (5%), with a specificity bias toward IFN-ω in APS-1 and toward IFN-α in MG (73). Collectively, neutralizing anti-IFN-I autoantibodies emerge across both genetic and acquired disorders that converge on thymus-centered failures of tolerance, spanning *AIRE* deficiency and related IEIs, as well as thymoma/MG-associated “sick thymus” states. Within this broader anti-IFN-I landscape, IFN-ω frequently emerges as a dominant or highly prevalent specificity, although the degree of subtype resolution varies across individual IEIs and acquired syndromes. (**Table 2**).

### 4.2 Systemic autoimmunity associated with IFN-ω signals

In contrast to type I interferon deficiency, which compromises antiviral defense, excessive or sustained IFN-I activity can itself be pathogenic, as illustrated by monogenic type I interferonopathies in which diverse upstream defects converge on persistent IFNAR signaling (11, 74). Notably, these disorders have been largely defined at the level of composite IFN-I activity rather than individual ligands (75, 76). This pathogenic framework also extends beyond classical interferonopathies. Monogenic forms of systemic lupus erythematosus (SLE), including disorders caused by gain-of-function variants in *TLR7* or *UNC93B1* and deficiency of *DNASE1L3*, further link dysregulated nucleic acid sensing or clearance to excessive IFN-I activity (77). Although most SLE is polygenic, these observations place lupus-spectrum disease within a broader continuum of IFN-I-driven inflammation and provide a clinically relevant context in which subtype-resolved IFN-I activity, including that of IFN-ω, can be examined (78–80). Emerging studies suggest that IFN-ω may be a measurable component of this broader IFN-I milieu. High-sensitivity multiplex protein profiling in SLE detected circulating IFN-ω together with multiple IFN-α subtypes, but not IFN-β, and showed that IFN-ω levels were associated with disease activity, whole blood ISG score, complement consumption, and autoantibody profiles (78). Consistent with this, oligonucleotide microarray analysis identified *IFNW1* among the transcripts upregulated in SLE, exceeding a twofold increase relative to healthy controls in 9 of 10 patients, with fold-changes ranging from 2.57 to 148.79 (79). Transcriptomic deconvolution further suggested compartment-specific enrichment. In discoid lupus skin lesions, the *IFNB1*-response signature was the strongest (Hedges’ *g* = 12.4), with *IFNW1* ranking second (*g* = 9.7), whereas in class III/IV lupus nephritis, *IFNW1*-response signatures ranked among the more prominent signals in both glomeruli (g=3.8; tied with IL-12) and tubulointerstitium (g=1.9; tied with IFNA2) (80). Together, these data support an association between IFN-ω and lupus-associated IFN-I programs, but do not yet establish whether IFN-ω exerts an independent pathogenic role distinct from the broader interferon network. This distinction remains clinically relevant because the IFN-I pathway in SLE has already been targeted at multiple levels, including neutralization of IFN-α ligands such as sifalimumab, blockade of IFNAR with anifrolumab, inhibition of downstream JAK-STAT signaling, and upstream targeting of pDCs such as anti-BDCA2 strategies (81). Within this therapeutic landscape, JNJ-55920839, a monoclonal antibody that neutralizes most IFN-α subtypes together with IFN-ω, but not IFN-β, provides proof of concept that combined IFN-α/ω blockade can suppress IFN-I pharmacodynamic activity in clinical settings (82, 83). (**Table 2**).

Beyond SLE, direct evidence for endogenous IFN-ω involvement in systemic autoimmunity remains limited. In dermatomyositis, *IFNW1* transcripts were coordinately upregulated with multiple IFN-α subtypes in affected skin, although their correlation with the overall IFN signature was not strong (84). More broadly, IFN-I activation has been reported across rheumatoid arthritis, Sjögren’s syndrome, systemic sclerosis, mixed connective tissue disease, antiphospholipid syndrome and undifferentiated connective tissue disease, yet most studies rely on composite IFN-I or ISG-based readouts rather than subtype-resolved measurement of endogenous IFN-ω (85–88). Taken together, available data associate IFN-ω with inflammatory IFN-I programs beyond antiviral disease, particularly in lupus-spectrum disorders, but its ligand-specific and causal contribution to systemic autoimmunity remains insufficiently resolved.

## 5 Discussion

Human IFN-ω has long been overshadowed by IFN-α and IFN-β, yet human “natural perturbations” now indicate that loss of IFN-ω activity can be clinically consequential in selected settings. Neutralizing anti-IFN-ω autoantibodies occur across multiple life-threatening viral infections, including cases with isolated IFN-ω neutralization, suggesting that its antiviral activity is not always fully compensated by other IFN-I ligands. However, this pattern is better interpreted as context-dependent incomplete compensation rather than as evidence for receptor exclusivity or a universally distinct signaling pathway. IFN-ω uses the shared IFNAR1/IFNAR2 complex and induces largely overlapping antiviral programs, while differences in cellular source, tissue distribution, production kinetics, local concentration, receptor-binding dynamics, and selective neutralization may limit ligand interchangeability *in vivo*. Anti-IFN-ω autoantibodies also represent a stable immunophenotype of thymus-centered tolerance failure, particularly in *AIRE* deficiency and related genetic or acquired disorders. Conversely, lupus-spectrum studies suggest that endogenous IFN-ω may contribute to broader pathogenic IFN-I programs, although its independent role remains unresolved.

Further progress requires a focused and prioritized research strategy. First, subtype-resolved assays are needed to quantify endogenous IFN-ω separately from individual IFN-α subtypes and IFN-β in blood and disease-relevant tissues. Second, direct comparisons under matched concentrations, exposure durations, cellular contexts, and infection models are required to distinguish quantitative differences in shared IFNAR signaling from genuinely ligand-selective effects. Third, ancestry-diverse genetic studies should evaluate rare *IFNW1* variants identified in patients with otherwise unexplained severe viral infections and determine whether any reproducibly impair IFN-ω expression, secretion, receptor signaling, or antiviral activity. Although *IFNW1* shows evidence of purifying selection, its constraint is not absolute, and current data establish neither a uniform global distribution nor clear geographic restriction of clinically relevant variants. Finally, prospective studies should determine whether IFN-ω or anti-IFN-ω autoantibody measurements improve risk stratification or guide therapeutic intervention. Together, these approaches should define the specific contexts in which IFN-ω is protective, pathogenic, or clinically actionable.
